# Public health responses to ageing in the face of demographic change

**DOI:** 10.2471/BLT.26.020226

**Published:** 2026-02-01

## Abstract

An innovative model of integrated care is helping countries respond to the challenges of population ageing. Gary Humphreys reports.

“She was sitting in her wheelchair in a dark room,” says Emmanuel Odhiambo, a community health worker in Siaya, western Kenya. He recalls meeting a 76-year-old woman in the nearby village of Ng’iya in January 2025. “She told me she’d had five children, but that three had moved to the city and the others never came to see her. She said she felt abandoned. She was clearly very depressed.”

The encounter left a mark on Odhiambo, not because it was unusual but because it was so typical. “More than eight in every ten older people live in rural or remote areas in Kenya,” he says. “They are often marginalized by our rapidly changing society, and a health system that is poorly equipped to provide the care they need.”

It is a scenario playing out globally as populations age. “By 2050, more than two billion people will be aged 60 years or older,” says Ritu Sadana, an expert on health systems and policy for healthy ageing at the World Health Organization (WHO). “Some 80% of those people will be living in low- and middle-income countries.”

Two trends are driving this phenomenon: falling total fertility rates and increasing life expectancy. 

At first glance, Kenya would appear not to be concerned, with only 4.7% of its 57 million people currently aged 60 or older. However, that proportion is projected to rise to 9.8% by 2050, overtaking the share of children aged under 5 years. At the same time, cultural shifts linked to urbanization are taking their toll. “Traditionally, older people in Kenya were respected community members and family caregiving was a valued responsibility,” explains Odhiambo. “These norms are increasingly under strain.”

According to Hyobum Jang, a medical officer focusing on long-term care in WHO’s Ageing and Health Unit, this too is a global phenomenon. “Traditional models of familial elder support are weakening as people migrate to cities and sociocultural norms change,” he says. “It’s something we’re seeing in countries across all WHO regions.” 

Mexico is a case in point. The country’s total fertility rate has fallen from around 6.8 in the 1970s to about 1.8 today, well below the 2.3 replacement level. “By mid-century, one in five Mexicans will be aged 65 or over, triple today’s proportion,” says Luis Miguel Gutiérrez Robledo, who leads the Public Policy Lab at Mexico’s Instituto Nacional de Geriatría (INGER) and the WHO Collaborating Centre on Integrated Care for Older People, where he focuses on developing and implementing person-centred, function-oriented care models.

While population ageing presents public health challenges globally, low- and middle-income countries face particular constraints. “Many health systems in low- and middle-income countries were initially designed with an emphasis on maternal and child health and infectious disease control,” says Sadana. “This brought significant gains, but it has also left gaps in services for older people. As populations age, we see a mismatch between health needs and service delivery. Older people often live with multiple chronic conditions and functional impairments in physical and mental capacities that aren’t well addressed by disease-specific, acute-care models.”

To address this mismatch, WHO is seeking to encourage a more holistic, integrated approach to health and social service provision under the Integrated Care for Older People (ICOPE) approach. 

The main aim of ICOPE is to support healthy ageing which the Organization defines as the process of developing and maintaining functional ability and intrinsic capacity across mobility, cognition, vision, hearing, and psychological well-being. 

“Maintaining intrinsic capacity is crucial because it affects an older person’s ability to connect and interact with the world around them and to continue to do the things they most value in life,” Sadana explains. 

Yuka Sumi, medical officer in WHO’s Ageing and Health Unit, was instrumental in developing an ICOPE Handbook and digital applications to support health workers, including many community workers unfamiliar with providing care for older people.

“Leveraging community-based care is key,” says Sumi. “Most older people wish to remain in their homes and communities, maintaining independence and social connections. Bringing services closer reduces barriers to access, especially in rural or underserved areas, and enables earlier detection and management of health and functional decline.”

Since 2017, a number of countries have adopted ICOPE, often embedding it in ageing policy and primary care practice. In Brazil, for example, ICOPE principles are being piloted in primary care settings through research collaborations, with growing interest in linking them to healthy ageing and noncommunicable disease initiatives. These efforts complement the country’s family health strategy, which provides a platform for community-based care. 

In China, cities including Beijing, Shanghai and Chengdu have integrated ICOPE into municipal ageing policies, in coordination with the provincial level long-term care insurance schemes, with community health workers conducting annual assessments and delivering personalized interventions such as physiotherapy and cognitive support

In India, WHO-supported pilots in several states have demonstrated the feasibility of ICOPE in both rural and urban settings, complementing the government’s National Programme for Health Care of the Elderly (NPHCE) and the expanding network of Health and Wellness Centres under the Ayushman Bharat universal health coverage initiative.

In Mexico, Gutiérrez Robledo has played a leading role in supporting ICOPE’s adoption. In collaboration with WHO and the Davos Alzheimer’s Collaborative project, INGER piloted ICOPE in six primary care sites in Mexico City, using digital cognitive assessments and biomarker testing to detect early signs of dementia, often before symptoms appear. “Combined with digital memory tests, biomarker tests help doctors to spot risks earlier and guide care,” Gutiérrez Robledo explains.

Additionally, an ICOPE pilot was launched in the Iztacalco district, Mexico City, in 2022, using a theory of change approach and collaborating with local health workers. Results from the pilot were encouraging. 

“We found that health workers were able to implement ICOPE without any trouble and that it enhanced patient care and supported maintenance of intrinsic capacity,” says Oscar Alfonso Rojas Calixto, who participated in the implementation of ICOPE as deputy director of epidemiology and medicine in Iztacalco. 

According to Rojas Calixto, the project is continuing in Iztacalco and being expanded to the Coyoacán district of the city. And it is sorely needed. “A quarter of the older people live in poverty and many lack access to health services,” says Rojas Calixto, who stresses that social isolation is a key concern. “Older people are isolated in and around cities just as much as they are in remote rural areas,” he says, and points to recent efforts to foster social connections in *Pilares* cultural centres, where older adults can share meals, socialize and access basic services.

In June 2025, President Claudia Sheinbaum launched *Salud casa por casa* (House-to-house health) which is scaling nationwide with around 20 000 health workers conducting in-home visits for older adults and people with disabilities. Aligned with ICOPE, *Salud casa por casa* focuses on preserving intrinsic capacity and using digital cognitive assessments and biomarker testing for early intervention. 

The plan is to assess all adults aged 65 and over annually through home visits. Gutiérrez Robledo welcomes the initiative but cautions that screening everyone over 65 may waste limited resources and argues for a more targeted approach.

In Kenya, Odhiambo is working to build capacity of community health promoters by training them in ICOPE, and is also encouraged by the progress being made. “Community-based ICOPE screening has raised awareness of healthy ageing among both community health promoters and residents, enabling earlier identification of declines in intrinsic capacity and more timely referrals,” he says. 

But challenges remain. “ICOPE has raised awareness and expectations, but these are often disappointed,” Odhiambo says. “Limited transport, especially for older adults with disabilities, complicates referrals. Once at the clinic, people face long queues and staff too busy to listen. Add high medical costs, stigma and ageism, and it’s easy to see why older people may feel discouraged. I’ve referred community members to facilities, only to find they lack the necessary services or equipment. It’s demoralizing and erodes community trust.”

Sumi acknowledges the significance of these challenges and stresses the need for strong health systems based on primary health care, including referral when needs are identified. “Comprehensive service delivery must include access to functioning secondary and tertiary care,” she says. “If the system isn’t there, referrals go nowhere.”

Sadana sums up: “As populations age, the message is clear: integrated, community-based care can help older people live longer, healthier and more independent lives. But for that promise to be realized, investments in people, health systems and communities must keep pace with changing demographics.”

**Figure:**
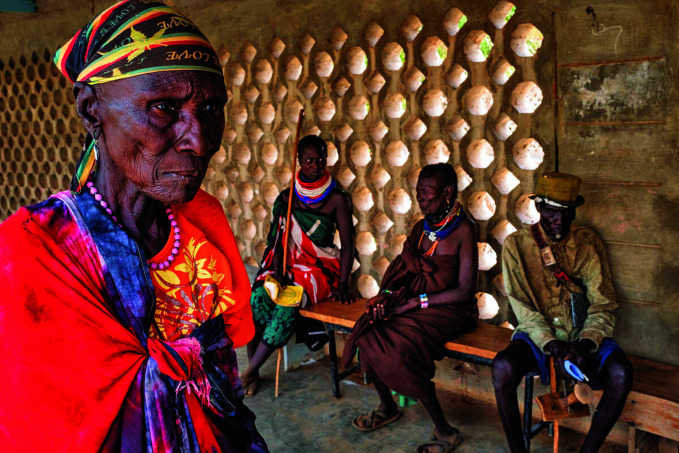
Patients waiting for cataract consultation in Kakuma Mission Hospital, Kenya.

WHO/NOOR/Sebastian Liste

**Figure:**
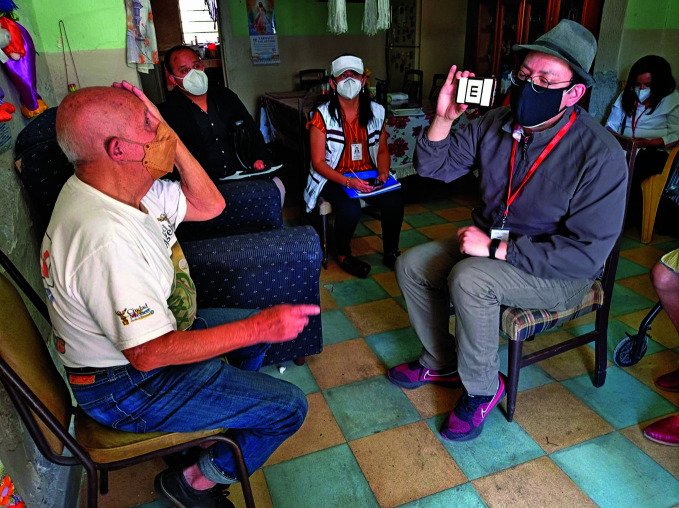
Sight testing as part of Integrated Care for Older People, Iztacalco district, Mexico City

Courtesy of Luis Miguel Gutiérrez Robledo

